# Locus coeruleus modulation of neurophysiological sensory selectivity differs in autism and other mental health conditions

**DOI:** 10.1038/s41398-026-03948-0

**Published:** 2026-03-24

**Authors:** Anna K. Müller, Christina Luckhardt, Christine M. Freitag, Nico Bast

**Affiliations:** Department of Child and Adolescent Psychiatry, Psychosomatics and Psychotherapy, University Medicine Frankfurt, Goethe-University, Deutschordenstraße 50, 60528 Frankfurt, Germany

**Keywords:** Neuroscience, Diagnostic markers, Autism spectrum disorders, Psychology

## Abstract

Sensory symptoms are common in autism and may result from differences in sensory processing. The locus coeruleus-norepinephrine (LC-NE) system modulates sensory processing by increasing selectivity to salience. To specify this mechanism, we manipulated LC-NE activity and explored sensory selectivity in autistic and non-autistic adolescents. We assessed pupillometry and electroencephalography (EEG) in 52 autistic adolescents (ASD), 55 healthy controls (CON), and 43 adolescents with other mental health conditions (MHC) during a passive auditory oddball task. A handgrip exercise manipulated LC-NE activity. Baseline pupil size (BPS) and stimulus-evoked pupillary response (SEPR) measured LC-NE tonic and phasic activity, respectively. Sensory selectivity was estimated with mismatch negativity (MMN) as change detection and P3a as bottom-up attention. Oddballs versus standards elicited increased SEPR, MMN amplitude, and longer MMN and P3a latencies. Increased P3a amplitude was more consistent in ASD. Across oddballs and standards, the manipulation transiently increased MMN and P3a amplitudes. A manipulation-induced BPS increase was specific to ASD and MHC. Pupillometric measures (BPS, SEPR) modulated sensory selectivity measures (MMN, P3a) differently between clinical groups (ASD, MHC) and CON. Findings indicate an altered orienting response to sensory stimuli in autism. The manipulation did not increase sensory selectivity but temporarily enhanced sensory reactivity to all stimuli with an arousal upregulation in both clinical groups. LC-NE activity was differentially related to sensory selectivity in autism and other mental health conditions. The tonic upregulation is discussed as increased stress susceptibility across autism and other mental health conditions.

## Introduction

Autism spectrum disorder (ASD) is a neurodevelopmental condition with core difficulties in social communication and interaction as well as repetitive and restricted behavior [1]. With DSM-5, sensory symptoms were introduced as a diagnostic criterion for autism. Sensory symptoms likely represent a different processing of sensory input [2, 3] that provoke behavioral responses like noise aversion [4]. Most autistic children report sensory symptoms [5]. However, underlying neurophysiological mechanisms remain poorly understood. Recently, the locus coeruleus-norepinephrine system (LC-NE) has been outlined as a key node in sensory processing [6, 7]. Here, we investigate LC-NE activity as a modulator of differences in sensory processing in autism and beyond.

The LC is a brainstem nucleus that synthesizes and proliferates cerebral norepinephrine (NE). Subcortical LC activity modulates sensory processing via cortical NE release at efferent target regions [8, 9]. Transient bursts of LC-NE phasic activity occur in response to a salient stimulus [10]. The associated NE release increases neuronal gain in synaptic signal transmission [11] predominantly for sensory processing networks [6, 12]. It represents an information filtering mechanism called sensory selectivity [13]. LC-NE tonic activity complements sensory selectivity as a slowly adapting baseline activity that fluctuates with arousal levels [14, 15]. Intermediate arousal levels are associated with the most pronounced LC-NE phasic activity and optimal task-related attentional performance [16, 17]. Within intermediate arousal levels, LC-NE tonic and phasic activity are inversely associated [18] and jointly coordinate selectivity in sensory processing.

Sensory processing is often investigated using an oddball paradigm in which a sequence of frequent standards is sometimes interrupted by a deviant oddball that induces sensory selectivity. Sensory symptoms in autism have been highlighted for the auditory domain [19], which suggest applying an auditory oddball task. A passive task further allows to investigate sensory selectivity independent of task-related executive functioning [10]. One study utilized a non-invasive handgrip exercise to experimentally manipulate LC-NE activity during an active auditory oddball task [20]. Applied between female participants only, the LC-NE manipulation decreased tonic activity and increased phasic activity which reflects enhanced sensory selectivity to salience. Increased activity in the frontoparietal attention network and decreased reaction times further suggest higher attentional performance.

In autism, we outlined LC-NE activity as a mechanism of sensory processing that modulated differences in social and non-social attention [21, 22]. However, different LC-NE functioning has also been associated with other mental health conditions, such as depression [23], anxiety [24] and attention deficit hyperactivity disorder (ADHD) [25]. This indicates differences in sensory processing in autism and also other mental health conditions, which may converge on an altered stress reactivity across mental health conditions [26].

Animal models established pupillometry as an indirect marker of LC-NE activity [18]. LC-NE activity indirectly inhibits pupil constriction via the parasympathetic Edinger Westphal nucleus and innervates sympathetic fibers eliciting pupil dilation [27]. Baseline pupil size (BPS) correlates with LC-NE tonic activity [28] while a stimulus-evoked pupillary response (SEPR) indicates LC-NE phasic activity [29]. An increased pupil response to non-social sounds has been shown to predict a later autism diagnosis in infants [30]. In autistic versus non-autistic preschoolers, a larger BPS was accompanied by attenuated SEPR during an auditory oddball paradigm [31]. These pupillometry findings indicate attenuated sensory selectivity in autistic children but remain correlative without an experimental manipulation of LC-NE activity.

Event-related potentials (ERPs) in electroencephalography (EEG) indicate cortical sensory selectivity during an oddball paradigm [32]. Mismatch negativity (MMN) is a negative ERP at around 150 ms that represents pre-attentive auditory change detection [33]. In autism, MMN has been associated with sensory over-responsivity [2] and sensory features [34]. However, a meta-analysis reported no differences in MMN amplitude to oddballs in autistic versus non-autistic children [35] and subsequent studies showed conflicting findings [36, 37]. A prolonged MMN latency in autistic children may indicate an increased processing time of change detection [37, 38]. The P3a is a positive ERP at around 250 ms that represents a bottom-up attentional mechanism in response to deviance [39]. We proposed LC-NE activity as a modulator of attentional function in autism, which suggests investigating effects of an LC-NE manipulation on the P3a [40]. However, meta-analyses reported no group differences in P3a amplitude or latency to oddball stimuli between autistic and non-autistic individuals [36, 41, 42]. Overall, cumulative evidence suggests no baseline alteration in P3a and MMN in autism. However, LC-NE activity manipulation might differently alter MMN and P3a between groups, which would indicate an altered LC-NE modulation of sensory selectivity.

Previous EEG studies on sensory processing in autism usually used aggregated data, which limits statistical power. In contrast, a single-trial level of analysis preserves trial-to-trial variability and enables to control for interindividual heterogeneity [43]. Such trial-level analyses showed an inverted U-shaped relationship of P3 amplitude and behavioral performance with BPS during an oddball task [44] and revealed BPS as a predictor of the P3b [45]. We utilize trial-level analyses to associate pupillometry with EEG.

This study aims to characterize sensory selectivity in autistic adolescents (ASD) compared to non-autistic controls (CON) and other mental health conditions (MHC). We apply pupillometry as a measure of subcortical LC-NE activity and EEG as a measure of cortical sensory selectivity while both are recorded during a passive auditory oddball task. A handgrip exercise manipulates LC-NE activity within subjects [20]. In autistic versus non-autistics, we expect higher BPS and attenuated SEPR. We hypothesize the manipulation to decrease BPS and increase SEPR across groups. The manipulation of BPS and SEPR is expected to emphasize MMN and P3a as improved sensory selectivity. These manipulation effects and their interactions are explored between groups. Findings could elucidate a neurophysiological pathway of different LC-NE modulation that explains altered sensory selectivity between different mental health conditions.

## Methods

### Sample

Recruitment was done through the outpatient clinic of the department of Child and Adolescent Psychiatry at the University Medicine of the Goethe University Frankfurt. The final sample (N = 150) consisted of 52 autistic adolescents (ASD), 55 healthy controls (CON), and 43 non-autistic adolescents with other mental health conditions (MHC). An autism diagnosis was based on DSM-V criteria confirmed by the Autism Diagnostic Interview (ADI-R) [46] and Autism Diagnostic Observation Schedule (ADOS version 1/2) [47]. The ASD group included 21 with no comorbidity, 16 with comorbid ADHD, five with comorbid anxiety disorders, one with comorbid depression; and 14 with other comorbidities. Participants in the CON group reported having no psychological or neurological diagnoses. The MHC group included primary diagnoses of depression (n = 16), anxiety disorder (n = 12), and ADHD (n = 15), of which 18 had additional comorbidities. Most participants were diagnosed at our university medicine during standardized procedures by trained clinicians; in a few cases, the diagnosis was self-reported based on previous assessments performed at other institutions. EEG data from nine participants and pupillometry data from two participants are missing due to technical failures. Some characteristics differed between groups (Table 1), which was considered in covariate analyses. All subjects reported unimpaired hearing. Exclusion criteria included neurological conditions or severe psychiatric comorbidity preventing EEG or pupillometry.

**Table 1:** Sample Description.

	ASD	CON	MHC	p value
n	52	55	43	NA
gender (f/m)	10/42	30/25	29/14	< 0.001
age	15.2 (2)	14.9 (1.8)	15.7 (1.9)	0.112
verbal IQ	97.3 (15.3)	105.8 (11.5)	101 (10.8)	< 0.001
non-verbal IQ	101.5 (14.8)	108 (11.2)	102.7 (11.8)	< 0.001
CBCL	65.9 (8.7)	50.5 (10.1)	61.5 (12.4)	< 0.001
YSR	63 (8.9)	55 (8.1)	65.7 (12)	< 0.001
SRS	24.9 (7.3)	5.6 (6.4)	11.7 (9.4)	< 0.001
SCQ	15.2 (4.7)	6.9 (4.2)	9 (5.5)	0.036
SP2 (auditory)	16.9 (8.4)	5.7 (5.7)	11.4 (10.1)	0.003
grip strength [z]	−1.8 (1.3)	−1.5 (1.4)	−1.1 (1.2)	0.022
included ET trials [%]	87 (12.1)	85.7 (18.3)	83.1 (19.6)	0.54
included EEG trials [%]	81.2 (24.2)	91.4 (14.8)	76.7 (35)	0.013
trial duration [s]	2.3 (0.4)	2.2 (0.4)	2.3 (0.6)	0.803

Sample size (n), gender (female/male), age (mean, standard deviation) and IQ (verbal, non-verbal) are depicted for ASD, CON and MHC group.

*ET* eye tracking, *EEG* electroencephalography.

Descriptive statistics (mean, standard deviation) for questionnaire data is provided. *CBCL* Child Behavior Checklist, *YSR* Youth Self-Seport, *SRS* Social Responsiveness Questionnaire, *SCQ* Social Communication Questionnaire, *SP2* Sensory Profile 2 (auditory subscale). Quantified grip strength during manipulation is shown as standardized values. Number of included trials for pupillometric and EEG data is reported in %. Trial duration (stimulus + ISI duration) is an inverse of central gaze stability in this gaze contingency task and is considered as a data quality measure that is depicted in s.

### Measures

Autism screening measures were applied with the Social Responsiveness Scale 2^nd^ edition [48] and Social Communication Questionnaire [49] to characterize autism symptoms between groups. In addition, the Sensory Profile 2 [50] was applied as a parent-report measure of sensory symptoms. Behavioral and emotional problems were assessed using the Child Behavior Checklist (CBCL 4–18) [51] as a caregiver report and the Youth Self-Report [52]. Four subtests of the Wechsler Intelligence Scale for either children (WISC-V) [53] or adults (WAIS-IV) [54] were applied to estimate verbal (similarities, vocabulary) and non-verbal (block design, matrix reasoning) IQ.

### Procedure

The study was approved by the ethical committee of the Medical Faculty of the Goethe University Frankfurt (20–1043). Written informed consent and assent was obtained. Participant compensation was 20 €. The study was carried out at our lab with controlled artificial lighting at 580 lux. The auditory oddball task was the first task of a two-task battery. We applied a concurrent EEG and eye-tracking recording during the tasks.

### Auditory oddball task

**Fig. 1:**
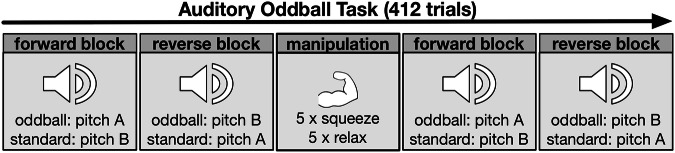
Auditory Oddball Task. The oddball task includes four task blocks à 103 trials. After the second block, a handgrip exercise aims to manipulate LC-NE activity. Pitch A/B are either 500 Hz or 750 Hz which is pseudo-randomized between subjects.

The passive auditory oddball task duration was 25 min (Fig. 1). Participants were seated with 65 cm distance in front of a 24-inch monitor (EIZO EV 2451, 1980 × 1080 pixels resolution). Pure tones were presented binaurally via over-ear headphones (Beyerdynamic DT 770 PRO) at 85 dB loudness. Participants were instructed to gaze at a centered, black fixation cross on a grey background and listen to the tone sequence. Trials were paused when gazes deviated from the fixation cross by more than 2.93 degrees of visual angle. In this case, a red rectangle around screen center accompanied by the message “eyes not detected” was presented to recenter visual attention.

The task consisted of four blocks. Each block included 100 pseudorandom pure tones (80% standards, 20% oddballs) preceded by three additional standard tones to establish standards. Each tone was presented for 100 ms and followed by a jittered interstimulus-interval between 1800 and 2000 ms. Oddball and standard tones differed in pitch (500/750 Hz). The initial oddball tone (500 or 750 Hz) was pseudo-randomly assigned between participants. Oddball tones were further counterbalanced within subjects between blocks before and after manipulation to control for possible frequency-specific effects (Fig. 1). The experimental manipulation was an isometric handgrip exercise (Mather et al., [20]) after the second block. The exercise asked participants to press a handgrip as hard as possible. A hand dynamometer quantified grip strength as a potential covariate. Grip strength was compared to norms [55] (Table 1). Within the experimental manipulation, a blue circle on a grey background prompted handgrip pressing for 18 s, followed by a yellow circle prompting resting for 60 s. This was repeated five times.

### Pupillometry

Pupillometric data were recorded at 300 Hz sampling rate using a Tobii Pro Spectrum (Tobii AB, Danderyd, Sweden). The eye tracker provides binocular pupil diameters using a 3D eye model. Prior to the experiment, a 6-point calibration process was performed using the Tobii Pro Eye Tracker Manager software (version 1.12.). The subject’s head could move freely, while a feedback signal indicated when head distance was outside the tracking space (distance: 55–75 cm, width: 42 cm, height: 26 cm). Pupillometric data were preprocessed with R (version 4.3.1.) following recent guidelines [56]. Raw data were segmented into trials. Implausible pupil diameter values (<2 mm, >8 mm) and dilation speed outliers (> 3 x median absolute deviation) were excluded. This step was repeated with the new trend line. We applied a blink identification for missing data within a 75–250 ms range and data points within 27 ms around blink gaps were discarded. Missing values up to 300 ms were imputed by linear interpolation. Pupil diameter values of both eyes were averaged. A subtractive baseline correction was applied [57]. For each trial, a baseline pupil size (BPS) was calculated as a mean of the pupil size during the first 250 ms after stimulus onset. Using a per-trial baseline captures changes within the task. This trial-baseline highly correlated with a distinct baseline estimation (gaze on grey screen for 5 s) before the oddball task (r = 0.8, p < 0.001) which emphasizes trial-based BPS as a valid baseline measure (see supplement 33). Stimulus-evoked pupillary response (SEPR) was defined as mean baseline-corrected pupil diameter during 500–1500 ms after stimulus onset. Pupil data preprocessing code is available here.

### Electroencephalography

Continuous 64-channel electroencephalography (EEG) was acquired using caps (Easycap GmbH, Herrsching, Germany) with sintered Ag/AgCl electrodes in an extended 10–20 layout. Electrodes placed at FCz and Afz served as reference and ground, respectively. Eye movements and blinks were recorded with an infraorbital electrode 1 cm below the right eye. Impedances were kept below 20 kΩ. Brain Vision Recorder software (version 1.25) with Brain Vision MR-Plus amplifiers (Brain Products GmbH, Gilching, Germany) recorded data at 1000 Hz sampling rate. An online anti-aliasing low-pass filter was applied with 0.01 Hz low cut-off and 250 Hz high cut-off. EEG data were processed off-line using Brain Vision Analyzer 2 software (version 2.3.0). All filtering was carried out with Butterworth zero-phase shift Filters. First, raw data were down-sampled to 500 Hz, re-referenced to average reference, and high-pass filtered at 1 Hz. During group-blind visual inspection, artefacts such as muscle activity were rejected. Bad channels were interpolated using spherical spline interpolation. Independent component analysis (ICA) was applied with the infomax algorithm. After removing artefactual ICA components (e. g. eye movements) based on two raters’ agreement, the ICA and inverse ICA transformation were applied to 0.1 Hz high-pass filtered data. The data was low-pass filtered at 30 Hz and segmented into trials including a pre-stimulus baseline of 500 ms. To ensure data quality, a second artifact rejection step was conducted through visual inspection to identify and remove any residual artifacts in the EEG data after the ICA. At trial level, MMN was defined as negative peak of a fronto-central electrode cluster (FC1, FC2, FCz, Fz) 100–150 ms after stimulus onset. At an aggregated level, additional MMN difference waves (oddball-standard) were calculated (supplements 13 + 14). P3a was defined as the positive peak of a central electrode cluster (Cz, FCz) 150–250 ms after stimulus onset. For both ERPs, mean amplitude ± 4 ms [µV] and latency [ms] were extracted.

### Statistical analysis

Statistical analysis was done in R version 4.3.1. (R Core Team, 2020) including additional packages (performance, lme4, lmerTest, emmeans, tidyverse, simr). For EEG, task blocks containing a minimum of 2/3 of data (13 oddball trials, 53 standard trials) were included. For pupillometry, trials with a minimum of 50% valid data points were included. Percentage of included trials are reported in Table 1, and per condition in supplement 30.

Linear mixed-effects models (LMM) were estimated using restricted maximum likelihood (REML) estimation for aggregated and trial level. Participant was always included as a random intercept. For aggregated-level analyses, measures were aggregated per condition and participant. For trial-level analyses, the same model structure was applied but measures were based on estimates per trial. Dependent variables were stimulus-evoked pupil response (SEPR), baseline pupil size (BPS), MMN and P3a amplitudes and latencies. Dependent variables were centered and scaled. Stimulus (oddball, standard), group (ASD, CON, MHC) and manipulation (before, after) were included as fixed effects with their interactions. We further included task block (forward, reverse) as additional fixed effect controlling for block-specific effects. All models included age and gender. We investigated stimulus and manipulation effects across and between groups. Subsequently, we investigated the association between the outcome variables. ERP variables were modelled with pupillometric metrics as predictors within the established fixed-effect structure of stimulus x group x manipulation x block.

Supplementary analyses include a model of MMN difference waves for amplitude and latency with group, manipulation and block as fixed effects. This allows to compare our single-trial analyses to aggregated MMN operationalizations [32, 35]. In covariate analyses on aggregated level, we investigated the effects of IQ, age and gender on dependent variables. We further investigated the influence of grip strength as effort on dependent variables in both task blocks after the manipulation.

All fixed effects and interactions were investigated with post-hoc contrasts of marginalized means. Effect sizes for LMMs are reported as marginalized fixed effects (β) for significant effects with 95% confidence intervals. Model fits are described by conditional R^2^ (cR^2^) and marginal R^2^ (mR^2^) [58]. Full models are shown in supplement 7–23. The analysis code is available here.

## Results

### Covariate effects

Grip strength did not influence manipulation-associated changes in any outcome variable (all p > 0.1, supplement 24–29). We explored IQ, age, and gender as covariates by investigating associations with the dependent variables (BPS, SEPR, MMN, P3a) across groups (supplement 1–6). We did not find an effect of verbal IQ or non-verbal IQ (p > 0.16). Age had an effect on BPS (F(1, 141) = 15.07, p < 0.001, mR^2^ = 0.14, cR^2^ = 0.91), MMN latency (F(1, 134) = 4.15, p < 0.05, mR^2^ = 0.06, cR^2^ = 0.48) and P3a amplitude (F(1, 134) = 10.72, p < 0.01, mR^2^ = 0.11, cR^2^ = 0.61). Age was associated with a decreased BPS (β = −0.16 [−0.25; −0.08]), shorter MMN latency (β = −0.07 [−0.14; −0.00]) and a smaller P3a amplitude (β = −0.12 [−0.19; −0.05]). Although gender was not significantly associated with the dependent outcome variables (all p > 0.1), it was retained in the models to adjust for imbalances in gender distribution. Thus, we controlled for age and gender in all subsequent models.

### Stimulus effects

**Fig. 2:**
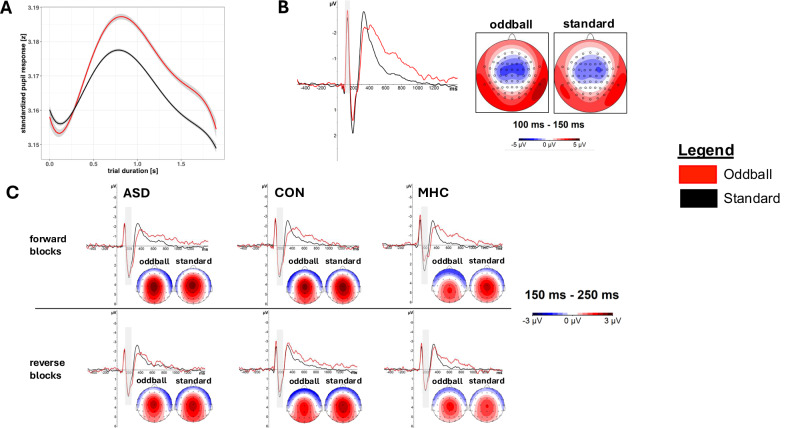
Stimulus Effect on SEPR, MMN and P3a. **A** Standardized pupil diameter changes by stimulus within trial averaged across conditions and subjects. Shaded area indicates 95% CI. Baseline pupil size (BPS) defined within 0–250 ms after stimulus onset; stimulus-evoked pupillary response (SEPR) defined within 500–1500 ms. **B** Grand average of pooled amplitude changes at electrodes FCz, Fz, FC1, FC2 within trial across all subjects including 500 ms pre-stimulus baseline. Grey area indicates MMN. Inlay: Topographies show amplitude voltages between 100–150 ms after stimulus onset by stimulus type across conditions and subjects. **C** Grand averages show pooled amplitude changes at electrodes Cz and FCz within trial averaged across conditions for ASD (left), CON (middle) and MHC group (right) including a 500 ms pre-stimulus baseline. Grey area indicates P3a. Inlay: Topographies depict amplitude voltages between 150–250 ms after stimulus onset.

Stimulus effects within trials are shown in Fig. 2. On aggregated level, SEPR differed by stimulus (F(1, 1015) = 26.78, p < 0.001, mR^2^ = 0.06, cR^2^ = 0.2) with a stronger pupillary response to oddballs versus standards (Δβ = 0.27 [0.17; 0.38]) (supplement 7). There was no stimulus effect on BPS (p > 0.8) (supplement 8). MMN amplitude differed by stimulus (F(1, 964) = 48.63, p < 0.001, mR^2^ = 0.04, cR^2^ = 0.65) with a more negative MMN amplitude to oddballs (Δβ = −0.25 [−0.33; −0.18]) (supplement 9). P3a amplitude was characterized by a three-way interaction of group, stimulus and block (F(2, 963) = 3.8, p < 0.05, mR^2^ = 0.10, cR^2^ = 0.61, supplement 10). In the forward blocks, the stimulus effect was significantly larger in the ASD than in the MHC group (Δβ = 0.51 [0.19; 0.84]), and larger in the CON compared to the MHC group (Δβ = 0.34 [0.02; 0.66]). No difference was found between ASD and CON group (Δβ = 0.18 [−0.12; 0.47]) or in reverse blocks (ASD-CON: Δβ = 0.26 [−0.04; 0.55], ASD-MHC: Δβ = 0.09 [−0.24; 0.41], CON-MHC: Δβ = −0.17 [−0.49; 0.15]). MMN latency did not differ by stimulus (p > 0.2) (supplement 11). P3a latency differed by stimulus (F(1, 963) = 16.56, p < 0.001, mR^2^ = 0.03, cR^2^ = 0.46) with a longer P3a latency to oddballs (Δβ = 0.18 [0.10; 0.27]) (supplement 12).

On trial level, the stimulus effects on SEPR, MMN amplitude, P3a amplitude and P3a latency were replicated (supplement 15, 16, 17). Additionally, a larger stimulus effect on P3a amplitude in the ASD versus the CON group was revealed in the reverse block (Δβ = 0.10 [0.01; 0.18]). Trial level analyses further revealed a stimulus effect on MMN latency (F(1, 50023) = 12.32, p < 0.001, mR^2^ = 0.004, cR^2^ = 0.05) with a longer MMN latency to oddballs (Δβ = 0.04 [0.02; 0.06]) (supplement 19).

### Manipulation effects

On aggregated level, the manipulation did not alter SEPR (p > 0.2; supplement 7). There was an interaction of manipulation and block on BPS (F(1, 1015) = 69.50, p < 0.001, mR^2^ = 0.14, cR^2^ = 0.91, supplement 8). Post-hoc contrasts indicated that this interaction is explained by a BPS increase in response to the manipulation in block 3 (Δβ = 0.38 [0.33; 0.43]) and block 4 (Δβ = 0.27 [0.22; 0.32]) versus block 2 (right before manipulation). Further, BPS decreased in block 2 versus block 1 (Δβ = −0.42 [−0.47; −0.37]) and in block 4 versus block 3 (Δβ = −0.11 [−0.16; −0.06]). Importantly, there also was an interaction of group and manipulation on BPS (F(2, 1015) = 11.48, p < 0.001, mR^2^ = 0.14, cR^2^ = 0.91). BPS increased after manipulation in ASD (Δβ = 0.15 [0.09; 0.21]) and MHC (Δβ = 0.20 [0.13; 0.27]) but not in CON (Δβ = −0.00 [−0.06; 0.05]). There was an interaction of manipulation and block on MMN amplitude (F(1, 964) = 5.75, p < 0.05, mR^2^ 0.04, cR^2^ = 0.65; supplement 9). Post-hoc contrasts indicated that this was explained by a more negative MMN amplitude in block 3 versus block 2 in response to the manipulation (Δβ = −0.13 [−0.23; −0.03]). This manipulation x block interaction was also found for P3a amplitude (F(1, 963) = 7.67, p < 0.01, mR^2^ = 0.10, cR^2^ = 0.61; supplement 10). This interaction was also explained by a more positive P3a amplitude only in block 3 versus block 2 in response to the manipulation (Δβ = 0.18 [0.07; 0.28]).

**Fig. 3:**
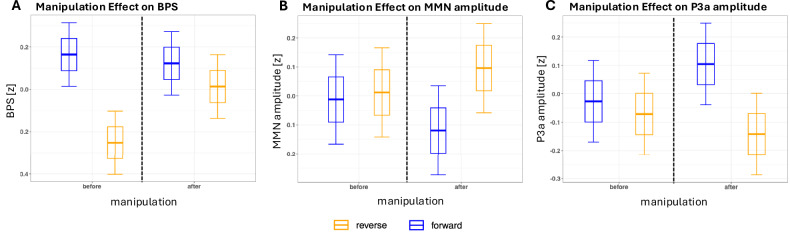
Manipulation Effect on BPS, MMN and P3a. Estimated marginal means (EMMs) of (**A**) standardized BPS (**B**) standardized MMN amplitude and (**C**) standardized P3a amplitude for each condition. Vertical dotted line indicates manipulation. Error bars show confidence intervals (95%). Boxes include data within 1 standard deviation.

On trial level, manipulation effects on BPS and P3a amplitude were replicated (supplement 17, 20). Figure 3 shows the specificity of the manipulation on BPS, MMN amplitude, P3a amplitude to the block right after the manipulation.

### Associations of the pupillometric measures

On trial level, BPS as predictor was linked to SEPR as dependent variable (F(1, 45612) = 3695, p < 0.001, mR^2^ = 0.17, cR^2^ = 0.31; supplement 21) with a negative association (β = −0.50 [−0.52; −0.48]). This linear association is supported by comparing it to non-linear associations based on exploratory model fit comparisons with Bayesian Information Criterion (BIC) (supplement 32). A three-way interaction with manipulation and block (F(1, 51062) = 16.61, p < 0.001, mR^2^ = 0.17, cR^2^ = 0.31) revealed a less negative association between BPS and SEPR after manipulation than before in reverse (Δβ = 0.07 [0.04; 0.10]) but not in forward blocks (Δβ = −0.02 [−0.05; 0.01]). This dynamic interaction of BPS and SEPR suggested considering both pupillometric variables in their association with ERPs.

### Associations of the pupillometric measures with ERPs

On trial level, SEPR as a predictor was significantly associated with MMN amplitude as a dependent variable (F(1,43842) = 72.83, p < 0.001, mR^2^ = 0.01, cR^2^ = 0.09; supplement 22), such that higher SEPR corresponded with less negative MMN amplitude (β = 0.05 [0.04; 0.06]). BPS was also associated with MMN amplitude (F(1,25047) = 4.75, p < 0.01, mR^2^ = 0.01, cR^2^ = 0.09). However, in contrast to SEPR, higher BPS was linked to more negative MMN amplitude (β = −0.02 [−0.04; −0.00]). Further, SEPR and BPS interacted with group in a three-way interaction on MMN amplitude (F(2, 43745) = 3.52, p < 0.05, mR^2^ = 0.01, cR^2^ = 0.09). At low BPS level (z = −2), the ASD group showed a significantly weaker association between SEPR and MMN amplitude compared to CON (Δβ = −0.08 [−0.16; −0.00]) and MHC (Δβ = −0.09 [−0.17; −0.00]), while CON and MHC did not differ (Δβ = −0.00 [−0.08; 0.07]). At both moderate and high BPS levels (z = 0, z = 2), groups did not significantly differ in the association between SEPR and MMN amplitude (all p < 0.05). Further, SEPR as predictor was linked to P3a amplitude as dependent variable (F(1, 43837) = 33.98, p < 0.001, mR^2^ = 0.03, cR^2^ = 0.14) by a positive association (β = 0.03 [0.02; 0.05], supplement 23). In addition, BPS interacted with group on P3a amplitude (F(2, 29969) = 4.95, p < 0.01, mR^2^ = 0.03, cR^2^ = 0.14). The CON group showed a positive association between BPS and P3a amplitude (β = 0.03 [0.01; 0.06]) while no association was found for the MHC group (β = −0.03 [−0.06; 0.00]) or for the ASD group (β = 0.02 [−0.01; 0.05]).

### Power simulation

Assuming a small effect size (β = 0.2) in our sample size (n = 150), the achieved power was 99.6% [98.98, 99.89] for detecting a significant interaction of group and manipulation based on Monte Carlo simulations, supplement 31.

## Discussion

The current study investigated sensory processing in autistic adolescents (ASD), non-autistic controls (CON) and other mental health conditions (MHC). We aimed to explore LC-NE activity as a modulator of cortical sensory selectivity by assessing concurrent pupillometry and EEG during a passive auditory oddball paradigm. A non-invasive manipulation of LC-NE activity was applied to investigate whether changes in subcortical LC-NE activity perpetuate to different cortical sensory selectivity between groups.

Across groups, sensory processing in response to salience (oddball versus standard tones) was characterized by increased LC-NE phasic activity (SEPR), increased pre-attentive change detection (MMN amplitude), and increased processing time (MMN and P3a latencies). Increased LC-NE phasic activity represents emphasized neuronal gain towards salience [11] that likely influence cortical sensory selectivity (MMN, P3a). Between groups, we observed an increased P3a amplitude towards oddballs versus standards in autistic individuals, whereas this stimulus effect was inconsistent in the CON and the MHC group. In autism, our findings suggest a neurophysiological bias towards salience during task-unrelated sensory processing. We did not find main effects of group on pupillometric measures (SEPR, BPS) in adolescents, which contrasts some previous findings in autistic versus non-autistic children [31] and infants [30]. We conclude that autistic differences in LC-NE activity may be specific to a younger age and compensate with development. Our null findings on MMN between autistic and non-autistic individuals are in line with meta-analytic findings on counterbalanced task design [32] and in autistic individuals with average cognitive ability [35]. We conclude that autistic and non-autistic individuals exhibit similar default LC-NE activity levels and pre-attentive auditory change detection but likely show a neurophysiological attention bias towards salience during task-unrelated sensory processing.

The modulation of this sensory processing was explored with an LC-NE manipulation within subjects. In a well-powered and transdiagnostic sample, we were not able to replicate the expected manipulation effects with BPS decrease and SEPR increase to oddballs [20]. The observed BPS upregulation, contrary to our hypothesis, may be influenced by currently unidentified factors, highlighting the need for further studies. However, we additionally assessed neurophysiological markers of cortical sensory processing which demonstrated an emphasized auditory change detection (MMN amplitude) and bottom-up attentional mechanism (P3a amplitude) after manipulation. Thus, the manipulation temporarily enhanced sensory reactivity. This manipulation effect was not specific to oddballs, which indicates increased sensory processing irrespective of stimulus salience. Enhanced neurophysiological measures of auditory sensory processing may explain faster reaction times in the auditory modality recently found using the same manipulation [59]. Our manipulation effects were specific to block 3 right after the manipulation suggesting that the erimentally increased sensory reactivity only lasted for around five minutes post manipulation across groups.

Importantly, the manipulation effect on BPS differed between groups, with a BPS increase specific to ASD and MHC. It suggests an LC-NE tonic upregulation post manipulation in the clinical groups, which might represent an arousal upregulation in response to a potential stressor [60]. The increased susceptibility to the manipulation in the ASD group may emerge from overall less stable neural dynamics recently found in autistic adolescents during resting-state EEG [61]. Increased LC-NE tonic activity favors unselective stimulus reactivity [28, 60] but likely does not increase sensory selectivity [16], which contrasts the original implementation [20]. This is plausible given the original implementation included an active task during magnet-resonance imaging, which might have been experienced as an arousing situation [62]. There, the manipulation could have acted as a stress relief that reduced arousal like progressive muscle relaxation. In our implementation, participants were presented with a passive task, in which arousal waned across groups (Fig. 3A). Here, the manipulation potentially acted as a relative stressor to the clinical groups that increased arousal. We propose this arousal upregulation as a transdiagnostic marker of increased susceptibility to stress, which is corroborated by animal models [26]. Stress susceptibility is likely modulated by LC-NE tonic functioning, as anxiety and stress-related disorders have been associated with LC volumes [63], and stress in rodents was modulated by the LC-NE [64]. LC-NE tonic upregulation in response to a potential stressor is suggested as a promising transdiagnostic biomarker of stress susceptibility that might contribute to mental health conditions [65].

Lastly, we explored the association of subcortical LC-NE activity (BPS, SEPR) and cortical sensory selectivity (MMN, P3a). Both LC-NE phasic and tonic activity uniquely modulate sensitivity to auditory change (MMN amplitude). Further, SEPR-modulated MMN amplitude differed between groups depending on the arousal level (BPS). At low arousal level, the ASD group presented a weaker association between SEPR and MMN amplitude compared to the CON and MHC groups. The handgrip manipulation may compensate for this reduced modulatory effect in the ASD group by the observed arousal upregulation. In addition, an association between BPS as arousal measure and P3a amplitude was found only for the control group (CON). This association has been observed before in healthy individuals for pre-stimulus BPS and a post-stimulus P3 [66]. These associations indicate a different arousal modulation of cortical sensory processing between non-clinical and clinical groups. We further replicate an association of pupillary response (SEPR) and P3a in a transdiagnostic sample that has previously been observed in non-clinical individuals [67]. This indicates an attention modulation via the LC-NE system across conditions of mental health. These associations of subcortical LC-NE activity (BPS, SEPR) with neurophysiological markers of cortical sensory selectivity contrasts previous null findings [45, 68] and might be attributed to the higher statistical power of our trial-level analysis.

Our study is limited as manipulation effects observed in block 3 (forward) compared to block 2 (reverse) are confounded by block position and oddball tone assignment. In addition, the autistic sample had an average cognitive ability and therefore was unrepresentative of the autistic population. Given the distinct genotypes and phenotypes within autism spectrum disorder (ASD), which vary in developmental trajectory and attainment, the observed effects may not generalize across phenotypic subgroups within the autism spectrum [69, 70]. Although neurophysiological mechanisms have been suggested to underlie sensory symptoms, demonstrating this link is often challenging for self-reported sensory measures, which rely on subjective evaluation and may introduce additional variance. In line with previous literature [19, 71], we only found a weak and non-significant association between the SP2 auditory subscale score and P3a amplitude (supplement 34). Overall, our findings may indicate a neurophysiological marker of a biased attentional switch towards salience in autistic versus non-autistic individuals, which might contribute to sensory symptoms. A manipulation of LC-NE activity emphasized sensory reactivity to all stimuli and specifically increased LC-NE tonic activity in both clinical groups. This arousal upregulation is a promising transdiagnostic marker of increased susceptibility to stress [26]. Further, LC-NE phasic activity differentially modulated auditory change detection between groups at low arousal level, while LC-NE tonic activity as arousal measure only modulated neurophysiological attention switch in healthy controls. These findings support LC-NE activity as a subcortical neuromodulator of cortical sensory selectivity that is altered in autism and in other mental health conditions.

## Supplementary information

Locus coeruleus modulation of neurophysiological sensory selectivity differs in autism and other mental health conditions

## Data Availability

Scripts for pupil data preprocessing and full analysis can be found at github, accessible via the link provided in the methods. All data supporting this study are described in the text and in the supplementary material. Due to ethical considerations and data privacy, the raw data that is tied to person identifiable information cannot be publicly shared.
