# Supplementary material for: Locus coeruleus modulation of neurophysiological sensory selectivity differs in autism and other mental health conditions
Source: Transl Psychiatry. 2026 Mar 24;16:200. doi: 10.1038/s41398-026-03948-0 (PMC13039879; doi:10.1038/s41398-026-03948-0)
Supplement: Supplementary file 1 — Locus coeruleus modulation of neurophysiological sensory selectivity differs in autism and other mental health conditions [file 41398_2026_3948_MOESM1_ESM.pdf]

Supplements

Locus coeruleus modulation of neurophysiological sensory selectivity differs in autism and other mental health conditions

2025-11-03

Contents

Abbreviations **2**

Covariates Models **3**

Supplement 1: Covariates effects on SEPR . . . 3
Supplement 2: Covariates effects on BPS . . . 3
Supplement 3: Covariates effects on MMN amplitude . . . 4
Supplement 4: Covariates effects on P3a amplitude . . . 4
Supplement 5: Covariates effects on MMN latency . . . 5
Supplement 6: Covariates effects on P3a latency . . . 5

Main Models on Aggregated Level **6**

Supplement 7: Model for SEPR . . . 6
Supplement 8: Model for BPS . . . 6
Supplement 9: Model for MMN amplitude . . . 7
Supplement 10: Model for P3a amplitude . . . 7
Supplement 11: Model for MMN latency . . . 8
Supplement 12: Model for P3a latency . . . 8
Supplement 13: MMN amplitude (difference wave) . . . 9
Supplement 14: MMN latency (difference wave) . . . 9

Main Models on Trial Level **10**

Supplement 15: Model for SEPR . . . 10
Supplement 16: Model for MMN amplitude . . . 10
Supplement 17: Model for P3a amplitude . . . 11
Supplement 18: Model for P3a latency . . . 11
Supplement 19: Model for MMN latency . . . 12
Supplement 20: Model for BPS . . . 12
Supplement 21: Associations of pupillometric measures . . . 13
Supplement 22: Associations between pupillometric measures-MMN amplitude . . . 14
Supplement 23: Associations between pupillometric measures-P3a amplitude . . . 15

Grip Strength Models **16**

Supplement 24: Grip strength on BPS . . . 16
Supplement 25: Grip strength on SEPR . . . 16
Supplement 26: Grip strength on MMN amplitude . . . 17
Supplement 27: Grip strength on P3a amplitude . . . 17
Supplement 28: Grip strength on MMN latency . . . 18
Supplement 29: Grip strength on P3a latency . . . 18

Miscellaneous **19**

Supplement 30: Number of included trials per condition . . . 19

Supplement 31: Post-hoc power analysis 20
Supplement 32: Exploratory model fit for BPS-SEPR association 21
Supplement 33: Correlation: block baseline-trial baseline 21
Supplement 34: Correlation Sensory Profile 2 (SP2)-P3a amplitude 22

Abbreviations

SEPR = stimulus-evoked pupillary response
BPS = baseline pupil size
MMN = mismatch negativity
ASD = autism spectrum disorder group
CON = control group
MHC = mental health condition group

Covariates Models

Supplement 1: Covariates effects on SEPR

	Sum Sq	Mean Sq	NumDF	DenDF	F value	Pr(>F)
stimulus	21.91	21.91	1	1015	26.78	0.000
manipulation	1.13	1.13	1	1015	1.38	0.241
group	2.53	1.27	2	141	1.55	0.216
block	6.19	6.19	1	1015	7.56	0.006
age	2.66	2.66	1	141	3.25	0.073
gender	0.01	0.01	1	141	0.01	0.905
verbal_IQ	0.22	0.22	1	141	0.27	0.607
non_verbal_IQ	0.16	0.16	1	141	0.20	0.654
stimulus:manipulation	1.12	1.12	1	1015	1.37	0.242
stimulus:group	0.15	0.08	2	1015	0.09	0.912
manipulation:group	0.55	0.28	2	1015	0.34	0.713
stimulus:block	1.66	1.66	1	1015	2.02	0.155
manipulation:block	2.85	2.85	1	1015	3.48	0.062
group:block	1.28	0.64	2	1015	0.78	0.457
stimulus:manipulation:group	1.54	0.77	2	1015	0.94	0.390
stimulus:manipulation:block	0.16	0.16	1	1015	0.20	0.659
stimulus:group:block	4.81	2.40	2	1015	2.94	0.054
manipulation:group:block	1.18	0.59	2	1015	0.72	0.486
stimulus:manipulation:group:block	2.98	1.49	2	1015	1.82	0.162

Supplement 2: Covariates effects on BPS

	Sum Sq	Mean Sq	NumDF	DenDF	F value	Pr(>F)
stimulus	0.00	0.00	1	1015	0.05	0.825
manipulation	3.81	3.81	1	1015	38.86	0.000
group	0.30	0.15	2	141	1.53	0.220
block	20.23	20.23	1	1015	206.54	0.000
age	1.48	1.48	1	141	15.07	0.000
gender	0.11	0.11	1	141	1.15	0.285
verbal_IQ	0.02	0.02	1	141	0.17	0.683
non_verbal_IQ	0.00	0.00	1	141	0.00	0.956
stimulus:manipulation	0.00	0.00	1	1015	0.00	0.969
stimulus:group	0.00	0.00	2	1015	0.02	0.978
manipulation:group	2.25	1.12	2	1015	11.48	0.000
stimulus:block	0.01	0.01	1	1015	0.15	0.702
manipulation:block	6.81	6.81	1	1015	69.50	0.000
group:block	0.22	0.11	2	1015	1.11	0.329
stimulus:manipulation:group	0.01	0.00	2	1015	0.03	0.974
stimulus:manipulation:block	0.01	0.01	1	1015	0.10	0.749
stimulus:group:block	0.01	0.00	2	1015	0.05	0.951
manipulation:group:block	0.02	0.01	2	1015	0.11	0.897
stimulus:manipulation:group:block	0.01	0.00	2	1015	0.03	0.971

Supplement 3: Covariates effects on MMN amplitude

	Sum Sq	Mean Sq	NumDF	DenDF	F value	Pr(>F)
stimulus	17.60	17.60	1	964	48.63	0.000
manipulation	0.05	0.05	1	964	0.13	0.718
group	0.28	0.14	2	134	0.39	0.681
block	3.75	3.75	1	964	10.37	0.001
age	0.04	0.04	1	134	0.11	0.737
gender	0.37	0.37	1	134	1.03	0.313
verbal_IQ	0.11	0.11	1	134	0.30	0.585
non_verbal_IQ	0.65	0.65	1	134	1.79	0.183
stimulus:manipulation	0.02	0.02	1	964	0.07	0.793
stimulus:group	0.90	0.45	2	964	1.24	0.290
manipulation:group	0.12	0.06	2	964	0.16	0.852
stimulus:block	0.00	0.00	1	964	0.00	0.966
manipulation:block	2.08	2.08	1	964	5.75	0.017
group:block	2.01	1.01	2	964	2.78	0.063
stimulus:manipulation:group	0.10	0.05	2	964	0.14	0.873
stimulus:manipulation:block	0.48	0.48	1	964	1.32	0.251
stimulus:group:block	1.21	0.61	2	964	1.68	0.187
manipulation:group:block	0.92	0.46	2	964	1.27	0.282
stimulus:manipulation:group:block	0.15	0.08	2	964	0.21	0.809

Supplement 4: Covariates effects on P3a amplitude

	Sum Sq	Mean Sq	NumDF	DenDF	F value	Pr(>F)
stimulus	6.26	6.26	1	963	15.43	0.000
manipulation	0.21	0.21	1	963	0.53	0.468
group	1.47	0.74	2	134	1.81	0.167
block	5.88	5.88	1	963	14.49	0.000
gender	0.92	0.92	1	134	2.26	0.135
age	4.35	4.35	1	134	10.72	0.001
verbal_IQ	0.03	0.03	1	134	0.08	0.777
non_verbal_IQ	0.62	0.62	1	134	1.52	0.220
stimulus:manipulation	0.04	0.04	1	963	0.09	0.762
stimulus:group	4.32	2.16	2	963	5.33	0.005
manipulation:group	0.03	0.01	2	963	0.04	0.965
stimulus:block	0.03	0.03	1	963	0.09	0.771
manipulation:block	3.11	3.11	1	963	7.67	0.006
group:block	1.47	0.74	2	963	1.81	0.164
stimulus:manipulation:group	0.06	0.03	2	963	0.08	0.924
stimulus:manipulation:block	0.03	0.03	1	963	0.07	0.787
stimulus:group:block	3.08	1.54	2	963	3.80	0.023
manipulation:group:block	1.32	0.66	2	963	1.62	0.198
stimulus:manipulation:group:block	1.28	0.64	2	963	1.58	0.206

Supplement 5: Covariates effects on MMN latency

	Sum Sq	Mean Sq	NumDF	DenDF	F value	Pr(>F)
stimulus	0.82	0.82	1	964	1.53	0.216
manipulation	0.25	0.25	1	964	0.47	0.493
group	0.68	0.34	2	134	0.64	0.531
block	0.01	0.01	1	964	0.01	0.915
gender	1.60	1.60	1	134	2.99	0.086
age	2.23	2.23	1	134	4.15	0.044
verbal_IQ	0.49	0.49	1	134	0.91	0.342
non_verbal_IQ	0.00	0.00	1	134	0.01	0.923
stimulus:manipulation	0.02	0.02	1	964	0.04	0.834
stimulus:group	0.04	0.02	2	964	0.04	0.963
manipulation:group	1.64	0.82	2	964	1.53	0.216
stimulus:block	0.37	0.37	1	964	0.68	0.409
manipulation:block	1.29	1.29	1	964	2.41	0.121
group:block	0.02	0.01	2	964	0.02	0.984
stimulus:manipulation:group	1.40	0.70	2	964	1.30	0.272
stimulus:manipulation:block	0.30	0.30	1	964	0.55	0.457
stimulus:group:block	1.03	0.52	2	964	0.96	0.383
manipulation:group:block	0.41	0.20	2	964	0.38	0.683
stimulus:manipulation:group:block	1.41	0.70	2	964	1.31	0.269

Supplement 6: Covariates effects on P3a latency

	Sum Sq	Mean Sq	NumDF	DenDF	F value	Pr(>F)
stimulus	9.27	9.27	1	963	16.56	0.000
manipulation	1.00	1.00	1	963	1.79	0.182
group	0.28	0.14	2	134	0.25	0.780
block	5.23	5.23	1	963	9.35	0.002
gender	1.61	1.61	1	134	2.87	0.092
age	0.29	0.29	1	134	0.52	0.473
verbal_IQ	0.18	0.18	1	134	0.32	0.573
non_verbal_IQ	0.21	0.21	1	134	0.38	0.538
stimulus:manipulation	0.07	0.07	1	963	0.12	0.733
stimulus:group	0.25	0.12	2	963	0.22	0.802
manipulation:group	0.02	0.01	2	963	0.02	0.978
stimulus:block	0.30	0.30	1	963	0.53	0.465
manipulation:block	0.73	0.73	1	963	1.31	0.253
group:block	1.96	0.98	2	963	1.75	0.174
stimulus:manipulation:group	0.29	0.14	2	963	0.26	0.773
stimulus:manipulation:block	0.05	0.05	1	963	0.08	0.775
stimulus:group:block	1.01	0.50	2	963	0.90	0.406
manipulation:group:block	0.29	0.14	2	963	0.26	0.774
stimulus:manipulation:group:block	0.90	0.45	2	963	0.81	0.446

Main Models on Aggregated Level

Supplement 7: Model for SEPR

	Sum Sq	Mean Sq	NumDF	DenDF	F value	Pr(>F)
stimulus	21.91	21.91	1	1015	26.78	0.000
manipulation	1.13	1.13	1	1015	1.38	0.241
group	3.46	1.73	2	143	2.12	0.124
block	6.19	6.19	1	1015	7.56	0.006
age	3.94	3.94	1	143	4.82	0.030
gender	0.01	0.01	1	143	0.02	0.900
stimulus:manipulation	1.12	1.12	1	1015	1.37	0.242
stimulus:group	0.15	0.08	2	1015	0.09	0.912
manipulation:group	0.55	0.28	2	1015	0.34	0.713
stimulus:block	1.66	1.66	1	1015	2.02	0.155
manipulation:block	2.85	2.85	1	1015	3.48	0.062
group:block	1.28	0.64	2	1015	0.78	0.457
stimulus:manipulation:group	1.54	0.77	2	1015	0.94	0.390
stimulus:manipulation:block	0.16	0.16	1	1015	0.20	0.659
stimulus:group:block	4.81	2.40	2	1015	2.94	0.054
manipulation:group:block	1.18	0.59	2	1015	0.72	0.486
stimulus:manipulation:group:block	2.98	1.49	2	1015	1.82	0.162

Supplement 8: Model for BPS

	Sum Sq	Mean Sq	NumDF	DenDF	F value	Pr(>F)
stimulus	0.00	0.00	1	1015	0.05	0.825
manipulation	3.81	3.81	1	1015	38.86	0.000
group	0.28	0.14	2	143	1.44	0.239
block	20.23	20.23	1	1015	206.54	0.000
age	1.56	1.56	1	143	15.95	0.000
gender	0.11	0.11	1	143	1.17	0.281
stimulus:manipulation	0.00	0.00	1	1015	0.00	0.969
stimulus:group	0.00	0.00	2	1015	0.02	0.978
manipulation:group	2.25	1.12	2	1015	11.48	0.000
stimulus:block	0.01	0.01	1	1015	0.15	0.702
manipulation:block	6.81	6.81	1	1015	69.50	0.000
group:block	0.22	0.11	2	1015	1.11	0.329
stimulus:manipulation:group	0.01	0.00	2	1015	0.03	0.974
stimulus:manipulation:block	0.01	0.01	1	1015	0.10	0.749
stimulus:group:block	0.01	0.00	2	1015	0.05	0.951
manipulation:group:block	0.02	0.01	2	1015	0.11	0.897
stimulus:manipulation:group:block	0.01	0.00	2	1015	0.03	0.971

Supplement 9: Model for MMN amplitude

	Sum Sq	Mean Sq	NumDF	DenDF	F value	Pr(>F)
stimulus	17.60	17.60	1	964	48.63	0.000
manipulation	0.05	0.05	1	964	0.13	0.718
group	0.21	0.11	2	136	0.29	0.748
block	3.75	3.75	1	964	10.37	0.001
age	0.14	0.14	1	136	0.39	0.531
gender	0.38	0.38	1	136	1.04	0.310
stimulus:manipulation	0.03	0.03	1	964	0.07	0.793
stimulus:group	0.90	0.45	2	964	1.24	0.290
manipulation:group	0.12	0.06	2	964	0.16	0.852
stimulus:block	0.00	0.00	1	964	0.00	0.966
manipulation:block	2.08	2.08	1	964	5.75	0.017
group:block	2.01	1.00	2	964	2.78	0.063
stimulus:manipulation:group	0.10	0.05	2	964	0.14	0.873
stimulus:manipulation:block	0.48	0.48	1	964	1.32	0.251
stimulus:group:block	1.21	0.61	2	964	1.68	0.187
manipulation:group:block	0.92	0.46	2	964	1.27	0.282
stimulus:manipulation:group:block	0.15	0.08	2	964	0.21	0.809

Supplement 10: Model for P3a amplitude

	Sum Sq	Mean Sq	NumDF	DenDF	F value	Pr(>F)
stimulus	6.26	6.26	1	963	15.43	0.000
manipulation	0.21	0.21	1	963	0.53	0.468
group	1.19	0.60	2	136	1.47	0.233
block	5.88	5.88	1	963	14.49	0.000
gender	0.93	0.93	1	136	2.28	0.133
age	3.61	3.61	1	136	8.90	0.003
stimulus:manipulation	0.04	0.04	1	963	0.09	0.762
stimulus:group	4.32	2.16	2	963	5.33	0.005
manipulation:group	0.03	0.01	2	963	0.04	0.965
stimulus:block	0.03	0.03	1	963	0.08	0.771
manipulation:block	3.11	3.11	1	963	7.67	0.006
group:block	1.47	0.74	2	963	1.81	0.164
stimulus:manipulation:group	0.06	0.03	2	963	0.08	0.924
stimulus:manipulation:block	0.03	0.03	1	963	0.07	0.787
stimulus:group:block	3.09	1.54	2	963	3.80	0.023
manipulation:group:block	1.32	0.66	2	963	1.62	0.198
stimulus:manipulation:group:block	1.28	0.64	2	963	1.58	0.206

Supplement 11: Model for MMN latency

	Sum Sq	Mean Sq	NumDF	DenDF	F value	Pr(>F)
stimulus	0.82	0.82	1	964	1.53	0.216
manipulation	0.25	0.25	1	964	0.47	0.493
group	0.48	0.24	2	136	0.45	0.638
block	0.01	0.01	1	964	0.01	0.914
gender	1.58	1.58	1	136	2.95	0.088
age	3.15	3.15	1	136	5.88	0.017
stimulus:manipulation	0.02	0.02	1	964	0.04	0.834
stimulus:group	0.04	0.02	2	964	0.04	0.963
manipulation:group	1.65	0.82	2	964	1.53	0.216
stimulus:block	0.37	0.37	1	964	0.68	0.409
manipulation:block	1.29	1.29	1	964	2.41	0.121
group:block	0.02	0.01	2	964	0.02	0.984
stimulus:manipulation:group	1.40	0.70	2	964	1.30	0.272
stimulus:manipulation:block	0.30	0.30	1	964	0.55	0.457
stimulus:group:block	1.03	0.52	2	964	0.96	0.383
manipulation:group:block	0.41	0.20	2	964	0.38	0.683
stimulus:manipulation:group:block	1.41	0.71	2	964	1.31	0.269

Supplement 12: Model for P3a latency

	Sum Sq	Mean Sq	NumDF	DenDF	F value	Pr(>F)
stimulus	9.27	9.27	1	963	16.56	0.000
manipulation	1.00	1.00	1	963	1.79	0.181
group	0.31	0.15	2	136	0.28	0.759
block	5.24	5.24	1	963	9.35	0.002
gender	1.61	1.61	1	136	2.88	0.092
age	0.36	0.36	1	136	0.65	0.422
stimulus:manipulation	0.07	0.07	1	963	0.12	0.733
stimulus:group	0.25	0.12	2	963	0.22	0.803
manipulation:group	0.02	0.01	2	963	0.02	0.978
stimulus:block	0.30	0.30	1	963	0.53	0.465
manipulation:block	0.73	0.73	1	963	1.31	0.252
group:block	1.96	0.98	2	963	1.75	0.174
stimulus:manipulation:group	0.29	0.14	2	963	0.26	0.773
stimulus:manipulation:block	0.05	0.05	1	963	0.08	0.775
stimulus:group:block	1.01	0.51	2	963	0.90	0.406
manipulation:group:block	0.29	0.14	2	963	0.26	0.774
stimulus:manipulation:group:block	0.90	0.45	2	963	0.81	0.447

Supplement 13: MMN amplitude (difference wave)

	Sum Sq	Mean Sq	NumDF	DenDF	F value	Pr(>F)
manipulation	0.00	0.00	1	404	0.00	0.959
group	0.43	0.22	2	135	0.23	0.791
block	0.80	0.80	1	401	0.87	0.352
gender	0.03	0.03	1	135	0.03	0.856
age	2.84	2.84	1	134	3.10	0.081
manipulation:group	1.15	0.58	2	404	0.63	0.533
manipulation:block	1.14	1.14	1	400	1.24	0.266
group:block	3.41	1.70	2	400	1.86	0.157
manipulation:group:block	1.07	0.54	2	400	0.59	0.557

Supplement 14: MMN latency (difference wave)

	Sum Sq	Mean Sq	NumDF	DenDF	F value	Pr(>F)
manipulation	0.06	0.06	1	402	0.06	0.807
group	0.55	0.27	2	133	0.30	0.744
block	0.14	0.14	1	399	0.15	0.696
gender	0.01	0.01	1	133	0.02	0.899
age	1.81	1.81	1	132	1.95	0.165
manipulation:group	0.71	0.36	2	402	0.38	0.681
manipulation:block	1.37	1.37	1	399	1.48	0.225
group:block	1.17	0.59	2	399	0.63	0.532
manipulation:group:block	2.91	1.46	2	399	1.58	0.208

Main Models on Trial Level

Supplement 15: Model for SEPR

	Sum Sq	Mean Sq	NumDF	DenDF	F value	Pr(>F)
stimulus	33.22	33.22	1	51067	33.46	0.000
manipulation	1.41	1.41	1	51124	1.42	0.234
group	4.37	2.18	2	176	2.20	0.114
block	9.50	9.50	1	51115	9.57	0.002
age	5.96	5.96	1	134	6.00	0.016
gender	0.01	0.01	1	133	0.01	0.925
stimulus:manipulation	1.44	1.44	1	51067	1.45	0.229
stimulus:group	0.13	0.07	2	51068	0.07	0.936
manipulation:group	0.33	0.17	2	51123	0.17	0.846
stimulus:block	2.18	2.18	1	51068	2.20	0.138
manipulation:block	3.47	3.47	1	51118	3.50	0.062
group:block	1.41	0.71	2	51115	0.71	0.492
stimulus:manipulation:group	1.50	0.75	2	51067	0.75	0.470
stimulus:manipulation:block	0.18	0.18	1	51068	0.18	0.673
stimulus:group:block	5.69	2.84	2	51068	2.87	0.057
manipulation:group:block	0.86	0.43	2	51116	0.44	0.647
stimulus:manipulation:group:block	4.15	2.07	2	51068	2.09	0.124

Supplement 16: Model for MMN amplitude

	Sum Sq	Mean Sq	NumDF	DenDF	F value	Pr(>F)
stimulus	14.05	14.05	1	50023	15.30	0.000
manipulation	0.54	0.54	1	50030	0.59	0.444
group	1.96	0.98	2	138	1.07	0.347
block	6.89	6.89	1	50026	7.50	0.006
age	0.47	0.47	1	134	0.52	0.473
gender	0.76	0.76	1	134	0.83	0.364
stimulus:manipulation	0.03	0.03	1	50023	0.04	0.847
stimulus:group	1.29	0.65	2	50023	0.70	0.495
manipulation:group	0.24	0.12	2	50030	0.13	0.876
stimulus:block	0.11	0.11	1	50023	0.12	0.730
manipulation:block	2.46	2.46	1	50025	2.68	0.102
group:block	3.96	1.98	2	50026	2.15	0.116
stimulus:manipulation:group	0.35	0.18	2	50023	0.19	0.826
stimulus:manipulation:block	0.80	0.80	1	50023	0.87	0.352
stimulus:group:block	2.81	1.41	2	50023	1.53	0.217
manipulation:group:block	2.02	1.01	2	50025	1.10	0.332
stimulus:manipulation:group:block	0.05	0.03	2	50023	0.03	0.971

Supplement 17: Model for P3a amplitude

	Sum Sq	Mean Sq	NumDF	DenDF	F value	Pr(>F)
stimulus	0.23	0.23	1	50021	0.27	0.605
manipulation	1.99	1.99	1	50026	2.27	0.132
group	2.54	1.27	2	137	1.46	0.237
block	21.26	21.26	1	50024	24.33	0.000
age	15.92	15.92	1	134	18.22	0.000
gender	1.79	1.79	1	134	2.05	0.155
stimulus:manipulation	0.01	0.01	1	50021	0.01	0.912
stimulus:group	9.54	4.77	2	50021	5.46	0.004
manipulation:group	0.41	0.20	2	50026	0.23	0.792
stimulus:block	0.19	0.19	1	50021	0.22	0.638
manipulation:block	7.14	7.14	1	50023	8.17	0.004
group:block	5.65	2.83	2	50024	3.23	0.039
stimulus:manipulation:group	0.18	0.09	2	50021	0.10	0.902
stimulus:manipulation:block	0.37	0.37	1	50021	0.42	0.517
stimulus:group:block	8.93	4.46	2	50021	5.11	0.006
manipulation:group:block	2.29	1.15	2	50023	1.31	0.270
stimulus:manipulation:group:block	0.07	0.04	2	50021	0.04	0.958

Supplement 18: Model for P3a latency

	Sum Sq	Mean Sq	NumDF	DenDF	F value	Pr(>F)
stimulus	37.56	37.56	1	50022	39.67	0.000
manipulation	0.22	0.22	1	50032	0.23	0.628
group	1.39	0.70	2	141	0.74	0.481
block	15.72	15.72	1	50027	16.60	0.000
age	0.18	0.18	1	134	0.19	0.660
gender	3.30	3.30	1	134	3.48	0.064
stimulus:manipulation	0.25	0.25	1	50022	0.27	0.607
stimulus:group	0.74	0.37	2	50022	0.39	0.677
manipulation:group	1.10	0.55	2	50033	0.58	0.560
stimulus:block	1.23	1.23	1	50022	1.30	0.254
manipulation:block	1.10	1.10	1	50026	1.16	0.281
group:block	0.07	0.04	2	50027	0.04	0.962
stimulus:manipulation:group	3.65	1.82	2	50022	1.93	0.146
stimulus:manipulation:block	0.17	0.17	1	50022	0.18	0.674
stimulus:group:block	0.11	0.06	2	50022	0.06	0.942
manipulation:group:block	0.34	0.17	2	50026	0.18	0.835
stimulus:manipulation:group:block	4.70	2.35	2	50022	2.48	0.084

Supplement 19: Model for MMN latency

	Sum Sq	Mean Sq	NumDF	DenDF	F value	Pr(>F)
stimulus	11.76	11.76	1	50023	12.32	0.000
manipulation	0.85	0.85	1	50035	0.89	0.346
group	0.80	0.40	2	142	0.42	0.658
block	2.80	2.80	1	50029	2.94	0.087
age	7.16	7.16	1	134	7.51	0.007
gender	0.50	0.50	1	134	0.52	0.472
stimulus:manipulation	0.04	0.04	1	50023	0.04	0.847
stimulus:group	1.90	0.95	2	50023	0.99	0.370
manipulation:group	0.55	0.28	2	50036	0.29	0.748
stimulus:block	1.20	1.20	1	50023	1.26	0.261
manipulation:block	3.71	3.71	1	50028	3.89	0.049
group:block	0.42	0.21	2	50029	0.22	0.803
stimulus:manipulation:group	0.08	0.04	2	50023	0.04	0.956
stimulus:manipulation:block	0.75	0.75	1	50023	0.78	0.377
stimulus:group:block	0.01	0.00	2	50023	0.00	0.995
manipulation:group:block	0.38	0.19	2	50028	0.20	0.819
stimulus:manipulation:group:block	3.56	1.78	2	50023	1.86	0.155

Supplement 20: Model for BPS

	Sum Sq	Mean Sq	NumDF	DenDF	F value	Pr(>F)
stimulus	0.10	0.10	1	56941	0.31	0.581
manipulation	99.71	99.71	1	56941	309.86	0.000
group	0.91	0.46	2	143	1.42	0.245
block	481.10	481.10	1	56941	1495.12	0.000
age	5.04	5.04	1	143	15.67	0.000
gender	0.37	0.37	1	143	1.14	0.286
stimulus:manipulation	0.00	0.00	1	56941	0.00	0.973
stimulus:group	0.07	0.03	2	56941	0.10	0.901
manipulation:group	54.38	27.19	2	56941	84.49	0.000
stimulus:block	0.28	0.28	1	56941	0.87	0.350
manipulation:block	173.65	173.65	1	56941	539.66	0.000
group:block	6.50	3.25	2	56941	10.09	0.000
stimulus:manipulation:group	0.16	0.08	2	56941	0.25	0.780
stimulus:manipulation:block	0.28	0.28	1	56941	0.86	0.354
stimulus:group:block	0.21	0.10	2	56941	0.33	0.722
manipulation:group:block	0.28	0.14	2	56941	0.44	0.644
stimulus:manipulation:group:block	0.17	0.08	2	56941	0.26	0.774

Supplement 21: Associations of pupillometric measures

	Sum Sq	Mean Sq	NumDF	DenDF	F value	Pr(>F)
z_rpd_low	3357.86	3357.86	1	45612	3694.88	0.000
stimulus	31.68	31.68	1	51038	34.86	0.000
manipulation	24.94	24.94	1	51074	27.45	0.000
group	2.99	1.50	2	138	1.65	0.197
block	186.13	186.13	1	51108	204.81	0.000
age	14.66	14.66	1	137	16.13	0.000
gender	0.84	0.84	1	136	0.93	0.337
z_rpd_low:stimulus	0.98	0.98	1	51038	1.08	0.298
z_rpd_low:manipulation	3.56	3.56	1	51064	3.92	0.048
stimulus:manipulation	1.33	1.33	1	51038	1.46	0.227
z_rpd_low:group	10.06	5.03	2	44443	5.54	0.004
stimulus:group	0.13	0.07	2	51038	0.07	0.930
manipulation:group	18.33	9.17	2	51075	10.09	0.000
z_rpd_low:block	13.73	13.73	1	51059	15.11	0.000
stimulus:block	1.94	1.94	1	51038	2.14	0.144
manipulation:block	76.00	76.00	1	51072	83.63	0.000
group:block	1.83	0.91	2	51114	1.00	0.366
z_rpd_low:stimulus:manipulation	1.15	1.15	1	51038	1.26	0.261
z_rpd_low:stimulus:group	0.52	0.26	2	51038	0.29	0.751
z_rpd_low:manipulation:group	4.44	2.22	2	51064	2.44	0.087
stimulus:manipulation:group	1.60	0.80	2	51038	0.88	0.416
z_rpd_low:stimulus:block	0.07	0.07	1	51038	0.08	0.780
z_rpd_low:manipulation:block	15.09	15.09	1	51062	16.61	0.000
stimulus:manipulation:block	0.01	0.01	1	51038	0.01	0.924
z_rpd_low:group:block	1.20	0.60	2	51058	0.66	0.517
stimulus:group:block	6.00	3.00	2	51038	3.30	0.037
manipulation:group:block	2.14	1.07	2	51075	1.18	0.308
z_rpd_low:stimulus:manipulation:group	1.60	0.80	2	51038	0.88	0.414
z_rpd_low:stimulus:manipulation:block	1.03	1.03	1	51038	1.13	0.288
z_rpd_low:stimulus:group:block	2.35	1.17	2	51038	1.29	0.275
z_rpd_low:manipulation:group:block	4.44	2.22	2	51063	2.44	0.087
stimulus:manipulation:group:block	5.34	2.67	2	51038	2.94	0.053
z_rpd_low:stimulus:manipulation:group:block	1.09	0.54	2	51038	0.60	0.549

Supplement 22: Associations between pupillometric measures-MMN amplitude

	Sum Sq	Mean Sq	NumDF	DenDF	F value	Pr(>F)
z__rpd	66.36	66.36	1	43842	72.83	0.000
z__rpd__low	4.33	4.33	1	25047	4.75	0.029
stimulus	16.38	16.38	1	43713	17.98	0.000
manipulation	0.31	0.31	1	43777	0.34	0.557
group	1.89	0.95	2	140	1.04	0.356
age	0.37	0.37	1	136	0.41	0.524
gender	0.87	0.87	1	135	0.96	0.329
z__rpd:z__rpd__low	0.06	0.06	1	43744	0.07	0.795
z__rpd:stimulus	0.00	0.00	1	43722	0.00	0.976
z__rpd__low:stimulus	0.20	0.20	1	43713	0.22	0.637
z__rpd:manipulation	2.76	2.76	1	43726	3.03	0.082
z__rpd__low:manipulation	0.06	0.06	1	43766	0.07	0.798
stimulus:manipulation	0.15	0.15	1	43712	0.16	0.686
z__rpd:group	4.47	2.24	2	43834	2.46	0.086
z__rpd__low:group	1.66	0.83	2	22462	0.91	0.401
stimulus:group	1.24	0.62	2	43713	0.68	0.506
manipulation:group	0.48	0.24	2	43776	0.26	0.770
z__rpd:z__rpd__low:stimulus	0.02	0.02	1	43719	0.03	0.871
z__rpd:z__rpd__low:manipulation	0.00	0.00	1	43723	0.00	0.954
z__rpd:stimulus:manipulation	0.07	0.07	1	43720	0.08	0.775
z__rpd__low:stimulus:manipulation	2.95	2.95	1	43712	3.24	0.072
z__rpd:z__rpd__low:group	6.41	3.21	2	43745	3.52	0.030
z__rpd:stimulus:group	1.65	0.83	2	43722	0.91	0.404
z__rpd__low:stimulus:group	2.73	1.36	2	43713	1.50	0.224
z__rpd:manipulation:group	1.96	0.98	2	43727	1.07	0.341
z__rpd__low:manipulation:group	0.83	0.42	2	43768	0.46	0.634
stimulus:manipulation:group	0.41	0.20	2	43712	0.22	0.800
z__rpd:z__rpd__low:stimulus:manipulation	0.07	0.07	1	43718	0.07	0.787
z__rpd:z__rpd__low:stimulus:group	3.76	1.88	2	43719	2.06	0.127
z__rpd:z__rpd__low:manipulation:group	0.24	0.12	2	43723	0.13	0.877
z__rpd:stimulus:manipulation:group	1.69	0.85	2	43721	0.93	0.395
z__rpd__low:stimulus:manipulation:group	1.07	0.54	2	43712	0.59	0.555
z__rpd:z__rpd__low:stimulus:manipulation:group	1.10	0.55	2	43718	0.60	0.548

Supplement 23: Associations between pupillometric measures-P3a amplitude

	Sum Sq	Mean Sq	NumDF	DenDF	F value	Pr(>F)
z__rpd	29.50	29.50	1	43837	33.98	0.000
z__rpd__low	0.61	0.61	1	32223	0.70	0.402
stimulus	0.36	0.36	1	43710	0.41	0.521
manipulation	3.80	3.80	1	43758	4.38	0.036
group	2.36	1.18	2	138	1.36	0.260
age	14.86	14.86	1	136	17.12	0.000
gender	1.67	1.67	1	134	1.92	0.168
z__rpd:z__rpd__low	0.37	0.37	1	43732	0.42	0.516
z__rpd:stimulus	0.13	0.13	1	43716	0.15	0.697
z__rpd__low:stimulus	0.01	0.01	1	43710	0.02	0.899
z__rpd:manipulation	1.60	1.60	1	43719	1.85	0.174
z__rpd__low:manipulation	1.88	1.88	1	43749	2.17	0.141
stimulus:manipulation	0.17	0.17	1	43709	0.19	0.659
z__rpd:group	1.24	0.62	2	43841	0.71	0.491
z__rpd__low:group	8.59	4.29	2	29969	4.95	0.007
stimulus:group	6.69	3.34	2	43710	3.85	0.021
manipulation:group	0.43	0.21	2	43758	0.24	0.783
z__rpd:z__rpd__low:stimulus	0.06	0.06	1	43714	0.07	0.787
z__rpd:z__rpd__low:manipulation	2.98	2.98	1	43717	3.44	0.064
z__rpd:stimulus:manipulation	0.39	0.39	1	43715	0.45	0.505
z__rpd__low:stimulus:manipulation	0.36	0.36	1	43710	0.41	0.520
z__rpd:z__rpd__low:group	0.26	0.13	2	43733	0.15	0.859
z__rpd:stimulus:group	0.13	0.06	2	43717	0.07	0.929
z__rpd__low:stimulus:group	4.88	2.44	2	43710	2.81	0.060
z__rpd:manipulation:group	0.77	0.39	2	43720	0.44	0.641
z__rpd__low:manipulation:group	5.10	2.55	2	43750	2.94	0.053
stimulus:manipulation:group	0.25	0.13	2	43709	0.15	0.865
z__rpd:z__rpd__low:stimulus:manipulation	1.48	1.48	1	43714	1.70	0.192
z__rpd:z__rpd__low:stimulus:group	5.19	2.60	2	43714	2.99	0.050
z__rpd:z__rpd__low:manipulation:group	0.28	0.14	2	43717	0.16	0.853
z__rpd:stimulus:manipulation:group	1.19	0.60	2	43715	0.69	0.504
z__rpd__low:stimulus:manipulation:group	0.08	0.04	2	43710	0.04	0.956
z__rpd:z__rpd__low:stimulus:manipulation:group	1.07	0.53	2	43714	0.62	0.540

Grip Strength Models

Supplement 24: Grip strength on BPS

	Sum Sq	Mean Sq	NumDF	DenDF	F value	Pr(>F)
stimulus	0.00	0.00	1	426	0.00	0.964
group	0.22	0.11	2	140	1.77	0.174
block	0.96	0.96	1	426	15.37	0.000
z__grip__strength	0.20	0.20	1	140	3.28	0.072
age	1.21	1.21	1	140	19.47	0.000
gender	0.04	0.04	1	140	0.67	0.414
stimulus:group	0.01	0.01	2	426	0.09	0.916
stimulus:block	0.01	0.01	1	426	0.12	0.724
group:block	0.09	0.05	2	426	0.75	0.472
stimulus:z__grip__strength	0.00	0.00	1	426	0.07	0.799
group:z__grip__strength	0.21	0.11	2	140	1.72	0.183
block:z__grip__strength	0.04	0.04	1	426	0.61	0.437
stimulus:group:block	0.00	0.00	2	426	0.01	0.990
stimulus:group:z__grip__strength	0.01	0.00	2	426	0.07	0.933
stimulus:block:z__grip__strength	0.00	0.00	1	426	0.01	0.916
group:block:z__grip__strength	0.11	0.06	2	426	0.89	0.412
stimulus:group:block:z__grip__strength	0.00	0.00	2	426	0.02	0.977

Supplement 25: Grip strength on SEPR

	Sum Sq	Mean Sq	NumDF	DenDF	F value	Pr(>F)
stimulus	3.90	3.90	1	426	4.42	0.036
group	0.68	0.34	2	140	0.39	0.680
block	0.56	0.56	1	426	0.63	0.426
z__grip__strength	3.11	3.11	1	140	3.53	0.062
age	6.77	6.77	1	140	7.68	0.006
gender	0.04	0.04	1	140	0.05	0.822
stimulus:group	1.19	0.60	2	426	0.68	0.509
stimulus:block	0.17	0.17	1	426	0.19	0.660
group:block	1.69	0.85	2	426	0.96	0.384
stimulus:z__grip__strength	0.20	0.20	1	426	0.23	0.635
group:z__grip__strength	0.02	0.01	2	140	0.01	0.987
block:z__grip__strength	0.37	0.37	1	426	0.42	0.517
stimulus:group:block	0.98	0.49	2	426	0.56	0.573
stimulus:group:z__grip__strength	0.88	0.44	2	426	0.50	0.607
stimulus:block:z__grip__strength	0.00	0.00	1	426	0.00	0.991
group:block:z__grip__strength	0.88	0.44	2	426	0.50	0.609
stimulus:group:block:z__grip__strength	0.01	0.00	2	426	0.00	0.996

Supplement 26: Grip strength on MMN amplitude

	Sum Sq	Mean Sq	NumDF	DenDF	F value	Pr(>F)
stimulus	4.41	4.41	1	403	11.64	0.001
group	0.43	0.22	2	133	0.57	0.567
block	3.23	3.23	1	403	8.51	0.004
z_grip_strength	0.00	0.00	1	133	0.00	0.988
age	0.05	0.05	1	133	0.13	0.721
gender	0.03	0.03	1	133	0.09	0.766
stimulus:group	0.37	0.19	2	403	0.49	0.614
stimulus:block	0.08	0.08	1	403	0.22	0.641
group:block	1.98	0.99	2	403	2.60	0.075
stimulus:z_grip_strength	0.06	0.06	1	403	0.16	0.688
group:z_grip_strength	0.17	0.08	2	133	0.22	0.802
block:z_grip_strength	0.22	0.22	1	403	0.59	0.443
stimulus:group:block	1.72	0.86	2	403	2.27	0.105
stimulus:group:z_grip_strength	1.06	0.53	2	403	1.39	0.250
stimulus:block:z_grip_strength	0.29	0.29	1	403	0.76	0.383
group:block:z_grip_strength	0.32	0.16	2	403	0.42	0.659
stimulus:group:block:z_grip_strength	1.41	0.71	2	403	1.86	0.157

Supplement 27: Grip strength on P3a amplitude

	Sum Sq	Mean Sq	NumDF	DenDF	F value	Pr(>F)
stimulus	0.08	0.08	1	402	0.22	0.636
group	0.42	0.21	2	133	0.61	0.546
block	6.56	6.56	1	402	18.90	0.000
z_grip_strength	0.96	0.96	1	133	2.77	0.098
age	2.61	2.61	1	133	7.51	0.007
gender	0.73	0.73	1	133	2.09	0.151
stimulus:group	0.57	0.29	2	402	0.83	0.439
stimulus:block	1.48	1.48	1	402	4.27	0.039
group:block	2.43	1.22	2	402	3.50	0.031
stimulus:z_grip_strength	0.91	0.91	1	403	2.63	0.106
group:z_grip_strength	0.04	0.02	2	133	0.06	0.939
block:z_grip_strength	0.76	0.76	1	403	2.18	0.141
stimulus:group:block	0.85	0.42	2	402	1.22	0.297
stimulus:group:z_grip_strength	0.32	0.16	2	402	0.46	0.635
stimulus:block:z_grip_strength	2.28	2.28	1	403	6.57	0.011
group:block:z_grip_strength	1.64	0.82	2	402	2.37	0.095
stimulus:group:block:z_grip_strength	0.17	0.09	2	402	0.25	0.777

Supplement 28: Grip strength on MMN latency

	Sum Sq	Mean Sq	NumDF	DenDF	F value	Pr(>F)
stimulus	0.96	0.96	1	403	1.86	0.174
group	0.05	0.03	2	133	0.05	0.951
block	0.00	0.00	1	403	0.00	0.982
z_grip_strength	0.00	0.00	1	133	0.01	0.931
age	2.24	2.24	1	133	4.36	0.039
gender	1.39	1.39	1	133	2.71	0.102
stimulus:group	0.84	0.42	2	403	0.82	0.443
stimulus:block	0.06	0.06	1	403	0.13	0.724
group:block	0.51	0.26	2	403	0.50	0.607
stimulus:z_grip_strength	0.49	0.49	1	403	0.95	0.331
group:z_grip_strength	0.57	0.28	2	133	0.55	0.578
block:z_grip_strength	0.77	0.77	1	403	1.49	0.222
stimulus:group:block	2.04	1.02	2	403	1.98	0.140
stimulus:group:z_grip_strength	0.77	0.38	2	403	0.74	0.476
stimulus:block:z_grip_strength	0.02	0.02	1	403	0.04	0.851
group:block:z_grip_strength	0.72	0.36	2	403	0.70	0.498
stimulus:group:block:z_grip_strength	3.00	1.50	2	403	2.92	0.055

Supplement 29: Grip strength on P3a latency

	Sum Sq	Mean Sq	NumDF	DenDF	F value	Pr(>F)
stimulus	1.40	1.40	1	402	2.77	0.097
group	0.04	0.02	2	133	0.04	0.963
block	3.52	3.52	1	402	6.96	0.009
z_grip_strength	0.23	0.23	1	134	0.46	0.498
age	0.00	0.00	1	133	0.00	0.979
gender	0.31	0.31	1	133	0.60	0.439
stimulus:group	0.13	0.07	2	402	0.13	0.876
stimulus:block	0.07	0.07	1	402	0.13	0.715
group:block	0.74	0.37	2	402	0.73	0.482
stimulus:z_grip_strength	0.03	0.03	1	403	0.06	0.800
group:z_grip_strength	0.60	0.30	2	134	0.59	0.556
block:z_grip_strength	0.68	0.68	1	403	1.35	0.247
stimulus:group:block	0.58	0.29	2	402	0.58	0.563
stimulus:group:z_grip_strength	0.02	0.01	2	403	0.02	0.980
stimulus:block:z_grip_strength	1.06	1.06	1	403	2.10	0.148
group:block:z_grip_strength	0.69	0.34	2	403	0.68	0.507
stimulus:group:block:z_grip_strength	1.44	0.72	2	403	1.42	0.243

Miscellaneous

Supplement 30: Number of included trials per condition

	Eye Tracking			EEG		
	ASD	CON	MHC	ASD	CON	MHC
before_forward_O500_S750	85.73 %	94.48 %	82.62 %	90.69 %	89.44 %	89.71 %
before_reverse_O750_S500	83.69 %	93.63 %	83.38 %	90.92 %	85.67 %	83.29 %
after_forward_O500S750	81.5 %	92.41 %	84.48 %	87.96 %	89.44 %	83.24 %
after_reverse_O750S500	80.35 %	91.93 %	80.24 %	84.38 %	86.81 %	78.33 %
before_forward_O750_S500	81.23 %	94.81 %	76 %	90.08 %	87.37 %	88.38 %
before_reverse_O500S750	77.69 %	93.22 %	73.38 %	82.58 %	87.19 %	84.71 %
after_forward_O750_S500	79.5 %	92.04 %	74.9 %	85.96 %	85.93 %	88 %
after_reverse_O500S750	80 %	91.93 %	73.1 %	83.04 %	86.37 %	85.24 %

"before_forward_O500_S750" as block 1, oddball 500Hz
"before_reverse_O750_S500" as block 2 oddball 750 Hz
"after_forward_O500S750" as block 3 oddball 500 Hz
"after_reverse_O750S500" as block 4 oddball 750 Hz
"before_forward_O750_S500" as block 1 oddball 750 Hz
"before_reverse_O500S750" as block 2 oddball 500 Hz
"after_forward_O750_S500" as block 3 oddball 750 Hz
"after_reverse_O500S750" as block 4 oddball 500 Hz

Supplement 31: Post-hoc power analysis

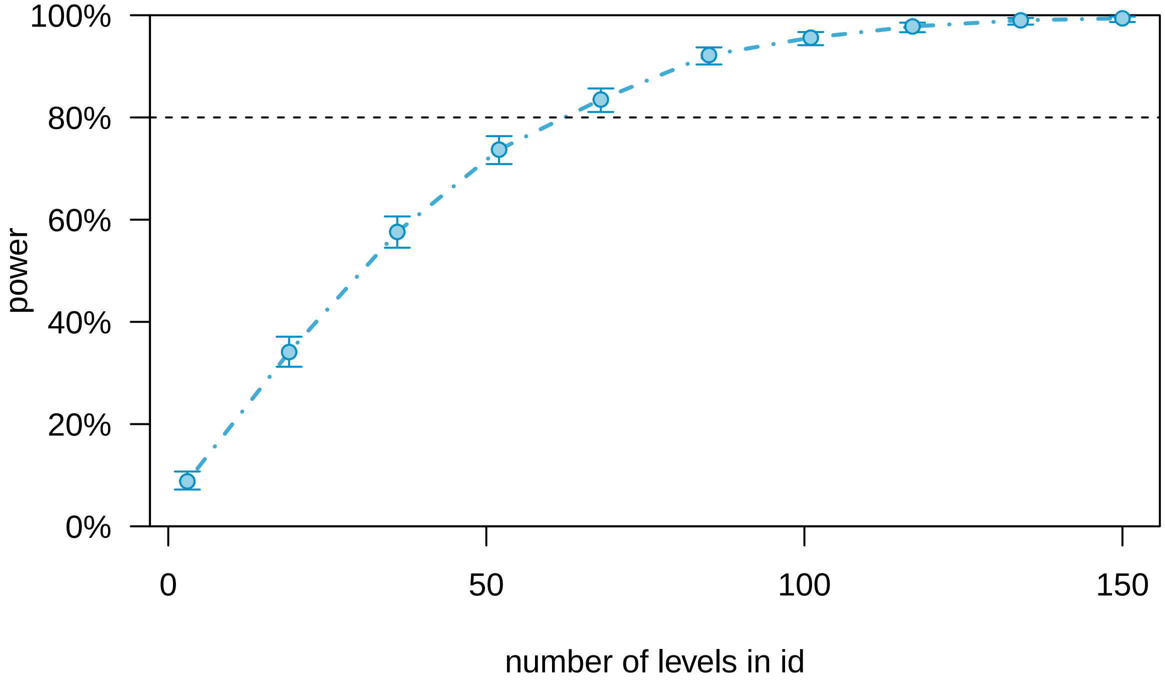

Power for model comparison, (95% confidence interval): 99.60% (98.98, 99.89)

Test: Likelihood ratio Comparison to y ~ group + condition + [re]

Based on 1000 simulations, (0 warnings, 0 errors) alpha = 0.05, nrow = 60000

Time elapsed: 0 h 12 m 10 s

Supplement 32: Exploratory model fit for BPS-SEPR association

	number of parameters	AIC	BIC	log likelihood	deviance	Chi-squared	df	p-value
linear_fit	52	141189.3	141649.2	-70542.66	141085.3	NA	NA	NA
quadratic_fit	76	141152.5	141824.6	-70500.23	141000.5	84.87118	24	0.0000000
cubic_fit	100	141144.8	142029.2	-70472.41	140944.8	55.62527	24	0.0002577

AIC = Akaike Information Criterion
BIS = Bayesian Information Criterion

Supplement 33: Correlation: block baseline-trial baseline

```
##         cor       p_value  conf_low conf_high
## 1 0.7983528 3.541106e-262 0.7766896 0.8181295
```

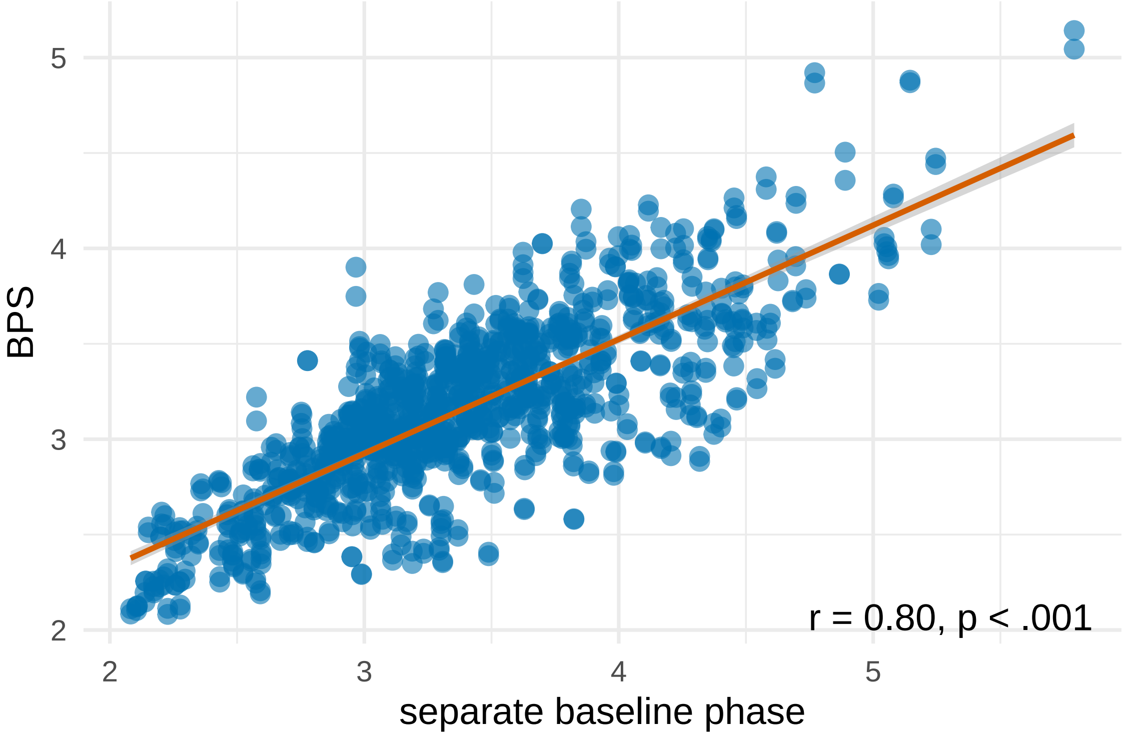

Supplement 34: Correlation Sensory Profile 2 (SP2)-P3a amplitude

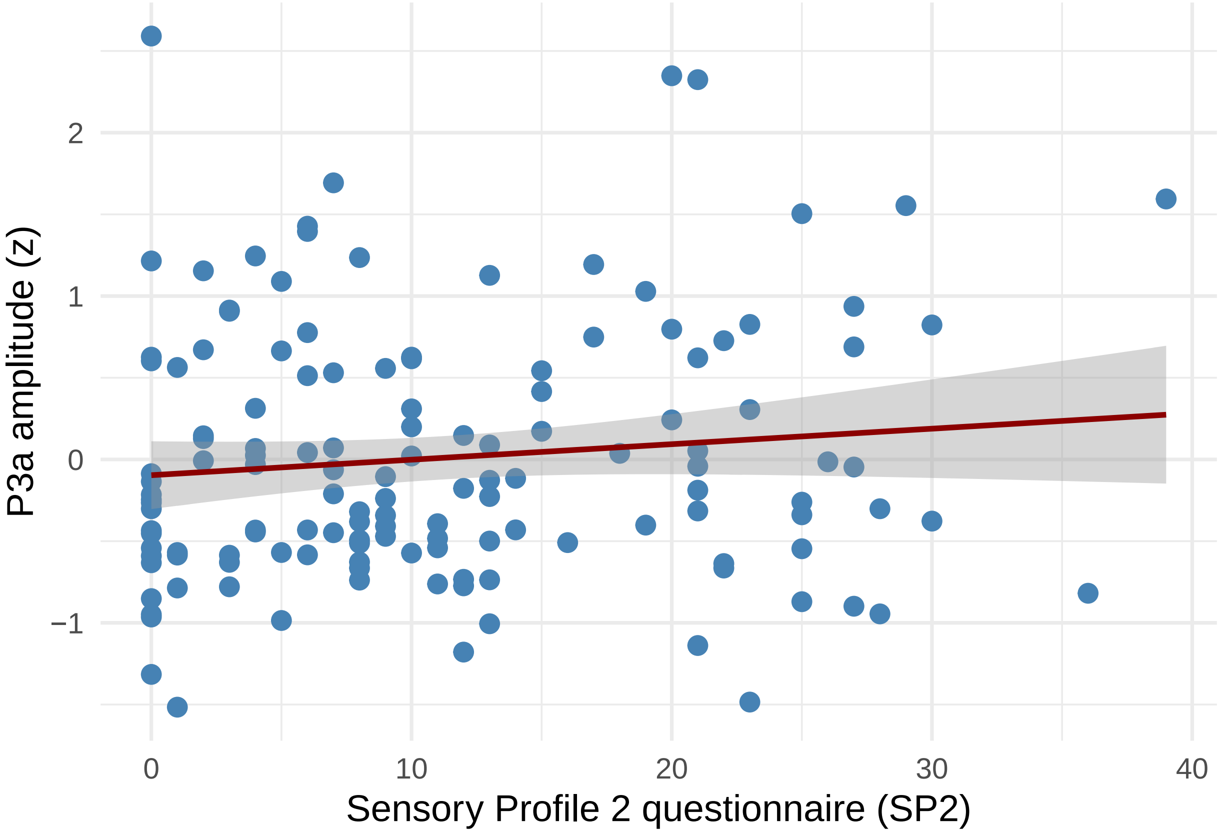


```
## # A tibble: 1 x 4
##     cor p_value conf_low conf_high
##   <dbl>   <dbl>    <dbl>     <dbl>
## 1 0.111   0.193  -0.0568     0.273
```
